# Assessing the Methylation Status of Two Potential Key Factors Involved in Cervical Oncogenesis

**DOI:** 10.3390/reports7030071

**Published:** 2024-08-18

**Authors:** Alina Fudulu, Marinela Bostan, Iulia Virginia Iancu, Adriana Pleșa, Adrian Albulescu, Irina Liviana Stoian, Demetra Gabriela Socolov, Gabriela Anton, Anca Botezatu

**Affiliations:** 1 Stefan S. Nicolau Institute of Virology, Romanian Academy, 030304 Bucharest, Romania; alina.fudulu@virology.ro (A.F.); iulia.iancu@virology.ro (I.V.I.); adriana.plesa@virology.ro (A.P.); adrian.albulescu@virology.ro (A.A.); gabi_anton2000@yahoo.com (G.A.); anca.botezatu@virology.ro (A.B.); 2 Department of Obstetrics and Gynecology, Grigore T. Popa University of Medicine and Pharmacy, 700115 Iasi, Romania; stoian.irinalv@yahoo.com (I.L.S.); demetrasocolov@gmail.com (D.G.S.)

**Keywords:** cervical cancer, DNA methylation, HPV infection, qRT-PCR

## Abstract

(1) Background: Cervical cancer, caused mainly by high-risk Human Papillomavirus (hrHPV), is a significant global health issue. While a Pap smear remains a reliable method for early detection, identifying new biomarkers to stratify the risk is crucial. For this purpose, extensive research has been conducted on detecting DNA methylation. (2) Methods: This cross-sectional study aimed to assess the expression levels of *EIF4G3* and *SF3B1* in precursor lesions and cervical tumor tissues through qRT-PCR and evaluate the methylation status of their promoters through bisulfite conversion. (3) Results: Both genes showed similar mRNA expression patterns, with the highest levels observed in squamous cell carcinoma (SCC) samples (*p* < 0.0001). Additionally, methylation analysis indicated increased percentages in the control group for both factors. Notably, the expression levels of both genes were inversely correlated with promoter methylation (*EIF4G3*—*p* = 0.0016; *SF3B1*—*p* < 0.0001). (4) Conclusions: Regarding the methylation pattern for both genes, we observe a decreasing trend from NILM to SCC patients. Therefore, we concluded that the decrease in methylation at the promoter level for both genes could be an indicator of abnormal cytology.

## 1. Introduction

Cervical cancer remains one of the most prevalent cancers among women globally and continues to pose a significant clinical and societal burden, particularly in resource-limited countries. According to the last statistics of GLOBOCAN from 2022, this is the fourth most common cancer among women (incidence of 6.8%), following breast, colorectal, and lung cancer. In addition, it is the most common cancer type in 25 countries and the leading cause of cancer death in 37 countries [1]. Cervical carcinogenesis progresses stepwise, beginning with HPV infection and advancing from premalignant stages to invasive cancer over at least a decade. Following a persistent infection with a hr-HPV, further viral-induced genetic and epigenetic alterations in the host cell genome are crucial for the development of cervical cancer [2]. 

Presently, the prevention and diagnosis of cervical cancer rely on cytological (Papanicolaou test) and histopathological examinations. The Pap smear is among the most reliable methods for early detection of cervical cancer (CC) and is considered the gold standard diagnostic test for asymptomatic women. In well-established healthcare systems, it can reduce the average annual mortality rate by 2.6% [3]. Some studies revealed a link between cervical cancer and some of its high-grade precursor lesions and elevated DNA methylation levels of numerous tumor suppressor genes [4]. Therefore, identifying new potential biomarkers to discern women at risk of developing cervical cancer is crucial. Furthermore, abnormal DNA methylation can occur in low-grade intraepithelial lesions (LGSIL), suggesting its potential application in the early diagnosis of cervical cancer. Detecting altered DNA methylation at this stage is very important, as LGSIL can either regress or progress to higher-grade lesions [5]. 

Currently, DNA methylation of the most investigated sites potentially associated with identifying high-grade cervical disease with good sensitivity and specificity includes *CADM1*, *MAL*, *miR-124a*, *EPB41L3*, *JAM3*, *TERT*, *C13ORF18*, *LMX1*, *SOX1*, *PAX1*, and *NKX6-1* [6,7,8]. Moreover, some of these genes are included in commercial DNA methylation tests, which offer the advantage of using tissue samples and other body fluids (e.g., liquid biopsy). In the context of cervical cancer, these assays can be used alone or combined with traditional screening methods to enhance triage and therapy management, providing valuable insights into epigenetic profiles [9]. 

Using ChIP-sequencing approaches, we previously identified nine genes as potential biomarkers, including *EIF4G3* (Eukaryotic translation initiation factor 4 γ 3) and *SF3B1* (Splicing factor 3b subunit 1) that are involved in similar putative pathways interacting with the same factors [10]. In this context, this study aimed to evaluate the potential of the investigated factors *EIF4G3* and *SF3B1*, including the methylation status of CpG islands around gene promoters and their expression in cervical cancer and precursor lesions, and to assess their prognostic potential.

## 2. Materials and Methods

### 2.1. Patients and Samples Collection

Cervical samples consisted of 52 HPV-positive cervical cytology specimens and tumor tissue from squamous cervical carcinomas (SCC) selected from a total of 110 women who self-referred for gynecological examinations “Cuza Voda” Clinical Hospital of Obstetrics and Gynecology, Iasi. The inclusion criteria for this study were women aged 18 and above who were not currently pregnant. Participants needed to have abstained from vaginal contact or showers for at least three days prior to sampling. The control group consisted of cervical specimens from women with negative cytology without HPV infection. This study followed the principles of the Declaration of Helsinki, and all participants provided written informed consent before taking part.

For the Papanicolaou and HPV genotyping test, a cervical sample was collected using a separate Cervex Brush (Avantor, Radnor, PA, USA). The liquid-based preparation method for collecting cervicovaginal samples was performed according to the manufacturer’s instructions (ThinPrep-Hologic, Bedford, MA, USA), and the samples were stored until further analysis. The samples were collected and preserved for DNA methylation analysis using an ESwab (COPAN, Brescia, Italy). (COPAN, Brescia, Italy). These specimens were stored at −80 °C until they were utilized. All samples for these assays were collected during a single visit.

Cytology diagnoses were made according to the Bethesda System grading criteria. Based on these management guidelines, selected patients were recommended to undergo colposcopy and either a Punch Biopsy (PB) or a Large Loop Excision of the Transformation Zone (LLETZ). Histology results were classified as no dysplasia, CIN grade I, II, III, or cervical cancer.

### 2.2. DNA Isolation

DNA was isolated from cervical specimens using the QIAamp DNA Mini Kit (Qiagen) following the producer’s guidelines. The concentration and purity of each DNA sample were assessed using a NanoDrop ND-1000 spectrophotometer (Thermo Fisher Scientific Inc., Waltham, MA, USA).

### 2.3. HPV DNA Detection and Genotyping

Human Papillomavirus (HPV) detection and genotyping were conducted for all samples using the INNO-LiPA^®^ HPV Genotyping Extra II kit (Fujirebio Europe, Ghent, Belgium) following the manufacturer’s instructions. The method allows the classification of samples into high-risk (hrHPV), low-risk (lrHPV), and undetermined-risk HPV types. The test specifically detects 13 high-risk HPV types: 16, 18, 31, 33, 35, 39, 45, 51, 52, 56, 58, 59, and 68.

### 2.4. Bisulfite Conversion

Bisulfite conversion was carried out using the EpiTect Bisulfite kit (Qiagen, Valencia, CA, USA) according to the manufacturer’s protocol. An input of 700 ng of the DNA sample in a total volume of 20 μL was converted, along with positive and negative controls (CpGenome Universal Methylated/Unmethylated DNA) (Millipore, Billerica, MA, USA).

### 2.5. RNA Isolation and cDNA Synthesis 

The extraction of total RNA was performed for all samples using TriZol reagent (Invitrogen, Carlsbad, CA, USA) and the purification with RNeasy Mini kit (Qiagen, Hilden, Germany) according to the manufacturer’s instructions. Total RNA samples were subsequently reverse-transcribed into cDNA utilizing the High-Capacity cDNA Reverse Transcription Kit (Thermo Fisher Scientific Inc.) using an input of 1 µg of each RNA sample.

### 2.6. Primer Design

Specific primers for the targeted genes were designed using the Primer-BLAST tool (www.ncbi.nlm.nih.gov/tools/primer-blast/, accessed on 23 February 2024). These qPCR data were analyzed, and relative expression was calculated using the quantification cycle (Cq) with the 2^−ΔCq^/2^−ΔΔCq^ method. Methylation primers were designed using the MethPrimer algorithm (http://www.urogene.org/methprimer/, accessed on 12 March 2024), which predicts CpG islands defined as 200 bp DNA sequences with a GC content greater than 50% [11]. The primers used in this study were synthesized by Biolegio (Nijmegen, The Netherlands). They were designed to distinguish between methylated and unmethylated DNA following bisulfite treatment. The sequences of all the primers used, along with their respective parameters, can be found in Table 1.

### 2.7. Quantitative Real-Time and Methylation-Specific Polymerase Chain Reaction (qRT-PCR and qMS-PCR)

For mRNA expression levels detection, qRT-PCR has been performed on Applied Biosystems 7300 Real-Time PCR system (Applied Biosystems, Foster City, CA, USA), using the GAPDH gene as a reference gene. The experiments were measured in triplicate, and relative expression was determined using the 2^−∆Cq^/2^−ΔΔCq^ method [12]. To assess the degree of methylation in the samples, direct quantitative methylation-specific PCR (qMSP) was performed on genomic DNA. Standard curves were created with serially diluted positive (fully methylated) and negative (fully unmethylated) controls at concentrations of 50 pg, 500 pg, 5 ng, and 50 ng. 

The qRT-PCR and qMS-PCR were conducted in a final volume of 25 µL, which included 12.5 µL of Maxima SYBR Green/ROX qPCR Master Mix (2X) (Thermo Fisher Scientific, Waltham, MA, USA), 0.30 µM for each primer, and 50 ng of target (cDNA, respectively bisulfite-treated DNA). The methylation percentage (%M) was calculated using the formula described by Fackler et al. (% methylation = 100 · [ng methylated gene A/(ng methylated gene A + ng unmethylated gene A)] [13]. The concentration of unmethylated (U) and methylated (M) DNA for each patient sample was extrapolated using the standard curves. 

### 2.8. Statistical Analysis 

The statistical analysis was performed with GraphPad Prism version 9.3 software (Graph Pad Software Inc., San Diego, CA, USA). To evaluate if the data sets were normally distributed, we applied the Shapiro–Wilk test, which is an appropriate method for small sample sizes (<50 samples). When *p* > 0.05, the null hypothesis is accepted, and data are called normally distributed. A simple linear regression test was used to evaluate the correlation between expression levels and gene methylation status. Moreover, the *t*-test parametric and Mann–Whitney non-parametric tests were used to compare study groups when appropriate. *p*-values < 0.05 were considered statistically significant. 

## 3. Results

### 3.1. Study Group Characterization

Samples from patients (*n* = 62) were divided into six groups according to their Papanicolaou test results and the presence or absence of HPV. The HPV-positive samples were classified in: 16.13% LGSIL (Low-Grade Squamous Intraepithelial Lesion) (*n* = 10), 16.3% HGSIL (High-Grade Squamous Intraepithelial Lesion) (*n* = 10), ASCUS (Atypical Squamous Cells of Undetermined Significance) (*n* = 10), 12.91% ASCH (Atypical Squamous Cells) (*n* = 8) and 22,58% tissue specimens from squamous cervical carcinomas (SCCs) (*n* = 14). The control group consisted of samples (16.13%) identified as Negative for Intraepithelial Lesion or Malignancy and negative for HPV (NILM−) (*n* = 10). In selected cases, targeted biopsies were performed following colposcopy for further evaluation. Notably, the biopsy results did not alter the LSIL, HGSIL cytological groups, and SCC classification. Regarding the ASCUS and ASCH groups, we selected the patients who underwent biopsies, and the histology results were more heterogeneous. The ASCUS patients included in this study presented 60% (6/10) CINI, 30% (3/10) CINII, and 10% CINIII. In the ASCH group, the most prevalent was CINIII diagnosis—50% (4/8), followed by CINII—37.5% (3/8), and CINI—12.5% (1/8).

**Figure 1:**
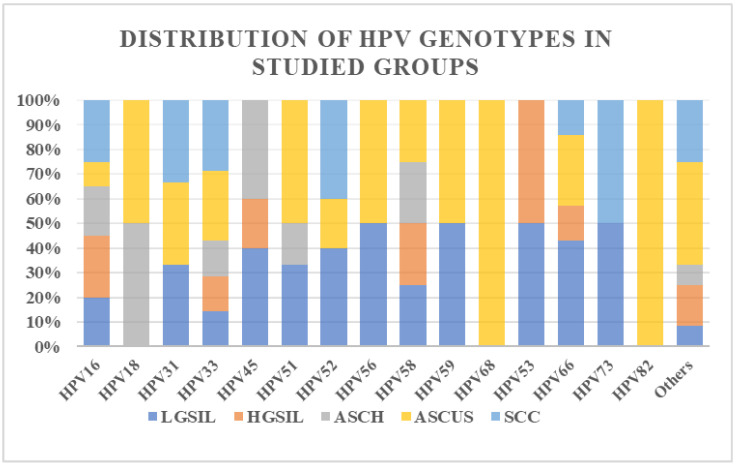
Distribution of HPV genotypes in studied groups according to cytology.

In terms of HPV genotype diversity, we observed that the LGSIL and AS-CUS groups showed the greatest variety, with over 10 genotypes present among the samples from these groups. In contrast, the SCC and HGSIL groups showed the least diversity, identifying only six genotypes. Notably, all patients in the SCC group had single HPV infections, specifically HPV16, HPV18, HPV45, and HPV52 (Figure 1). 

**Figure 2:**
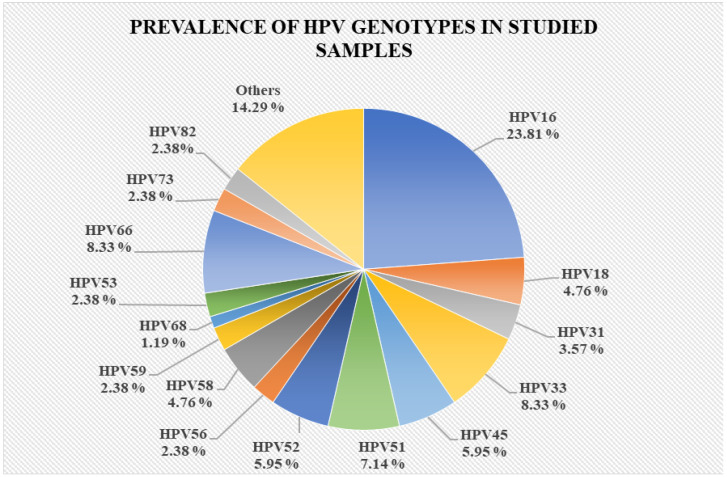
Prevalence of HPV genotypes in all studied samples.

Furthermore, we observed that HPV16 had the highest prevalence, accounting for 23.81% of the total investigated samples. Prevalence data are shown in Figure 2. 

### 3.2. Evaluation of Promoter Methylation Status

Upon evaluating the distribution of our data sets from the studied groups using the Shapiro–Wilk normality test, we found that all values for *EIF4G3* were normally distributed except in the SCC group. For *SF3B1*, the SCC and HGSIL groups deviated from normality. In these instances, the Mann–Whitney test was applied (Supplementary Table S1). When investigating the methylation status of both studied genes, we found that the highest percentage of methylation was in control samples, with medians of 88.76% (range: 81.20–98.35%) for *EIF4G3* and 87.53% (range: 78.95–98.22%) for *SF3B1*. In the *EIF4G3* promoter, CpG islands showed increased methylation percentages in ASCUS and LGSIL patients, with medians of 42.83% and 36.23%, respectively, but still lower than the control group. Samples from ASCH and HGSIL exhibited similar patterns, with median values of 13.22% and 15.21%, respectively, and methylation percentages ranging from 3.31 to 25.57% and 6.39 to 22.56%. The lowest values were observed in SCC tissue samples, with a median of 1.49% Table 2.

For the *SF3B1* promoter, the lowest methylation values were found in SCC samples (0–3.08%), followed by slight increases in the HGSIL (range: 38.61–73.89%) and ASCH (6.43–16.50%) groups. Higher percentages were observed in the LGSIL (15.97–36.60%) and ASCUS (38.61–73.89%) lesions.

**Figure 3:**
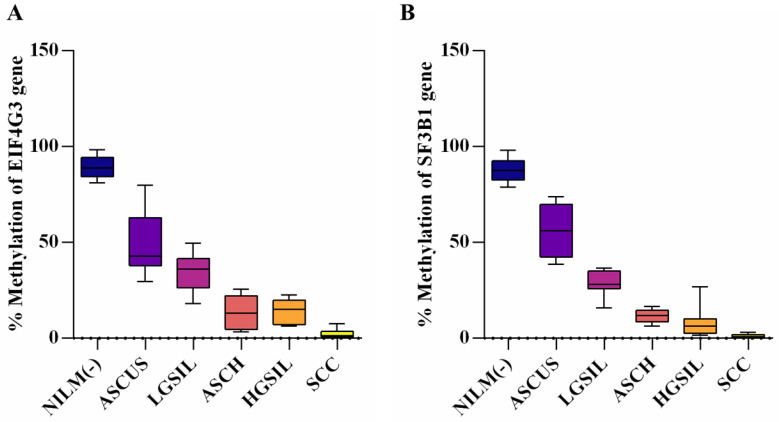
Methylation profiles in all studied groups for both EIF4G3 (**A**) and SF3B1 (**B**) genes.

All results indicate a significantly decreased percentage of promoter methylation in all studied groups compared with controls (*p* < 0.0001). The methylation profiles of *EIF4G3* and *SF3B1* in the studied groups are presented in Figure 3.

For both genes, a comparison of methylation levels between ASCUS and HGSIL showed a significant difference, with lower levels observed in HGSIL patients (*p* < 0.0001). Additionally, comparing the ASCH group with the SCC group revealed significant differences for both *EIF4G3* (*p* = 0.0004) and *SF3B1* (*p* < 0.0001). However, no statistical difference was found between the ASCH and HGSIL groups. The biopsy results showed that 60% of patients with ASCH presented a CINIII diagnosis, with this group presenting a similar percent of methylation level with HGSIL. 

### 3.3. Evaluation of Gene Expression Levels in Patient Samples and Correlation between Expression Levels and Methylation Percentage

After evaluating the distribution of gene expression values across the studied groups using the Shapiro–Wilk normality test, we found that all values adhered to a normal distribution (Supplementary Table S1). The qRT-PCR results indicated that mRNA expression levels of both *EIF4G3* and *SF3B1* genes were significantly higher in all studied groups compared with the control group, except for the ASCH group. Moreover, the SCC group exhibited the most significant increases for both genes (*p* < 0.0001), with mean values of −2.227 and −1.540, respectively, compared with the NILM (−) group, which had mean values of −4.649 and −4.242. Additionally, significant results were observed for the *EIF4G3* gene in the ASCUS group (*p* < 0.0001, median = −2.221) and for the *SF3B1* gene in the LGSIL group (*p* < 0.0001, median = −2.220) when compared with the control group Table 3.

**Figure 4:**
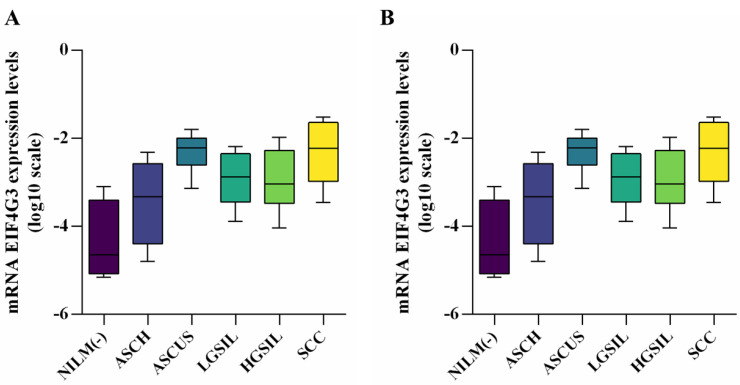
mRNA expression levels of *EIF4G3* (**A**) and *SF3B1* (**B**) gene promoters in studied groups.

The gene expression profiles of *EIF4G3* and *SF3B1* in the studied groups are presented in Figure 4.

When comparing the expression levels between the ASCH and SCC groups, there was a significantly higher expression of both genes in SCC patients (*p* = 0.0102 for *EIF4G3* and *p* < 0.0001 for *SF3B1*). In the comparison between the ASCH and HGSIL groups, a significant increase in expression was observed for the *SF3B1* gene in the HGSIL group. Conversely, when comparing the ASCUS and HGSIL groups, only *EIF4G3* expression levels were significantly higher in HGSIL patients (*p* = 0.0033). It seems that the expression level of the *SF3B1* gene could better discriminate between the ASCH and SCC groups. 

**Figure 5:**
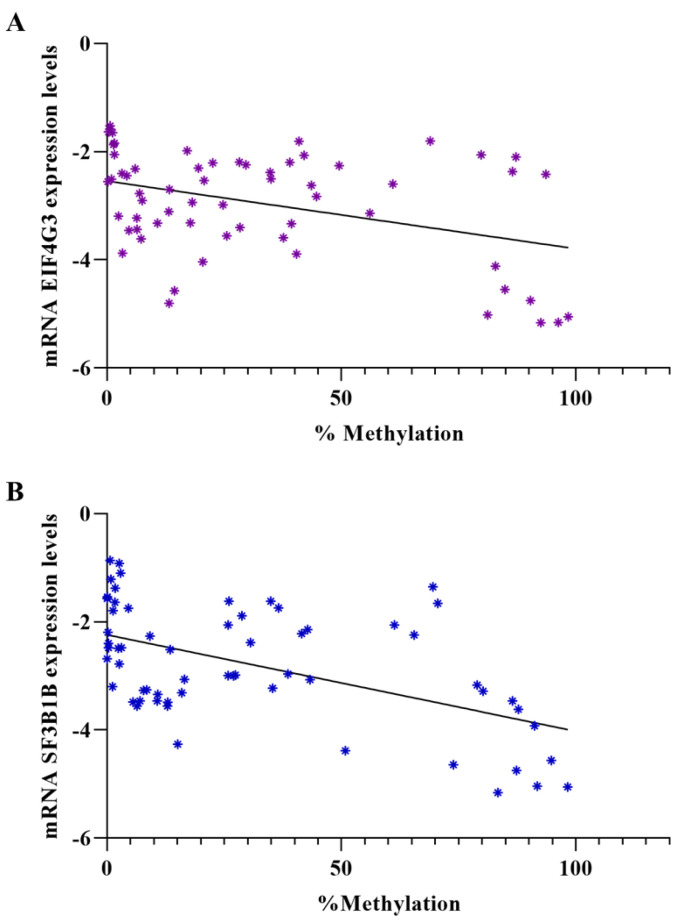
Correlation between mRNA expression levels and methylation status of promotors of EIF4G3 (**A**) and SF3B1 (**B**) genes (The asterisks indicate the points where the two coordinates—expression level and methylation percentage—intersect for a given sample. The colors are not significant).

Further, we investigated the correlation between mRNA expression levels and methylation status for both genes and observed a significant inverse correlation. For *EIF4G3*, the correlation had a *p*-value of 0.0016 (Y = −0.01252X − 2.543), and for *SF3B1*, the *p*-value was less than 0.0001 (Y = −0.01782X − 2.240) (Figure 5). 

## 4. Discussion

Testing for high-risk HPV (hrHPV) DNA with new molecular instruments demonstrates excellent performance and reproducibility. Cuzick et al. show that HPV testing has a sensitivity of 90–100% for detecting precancerous lesions, compared with a sensitivity of 50–80% for cytological screening [14,15]. Research in the field of epigenetics has demonstrated that aberrant DNA methylation is a common alteration in cancer [16]. The hypermethylation of specific DNA regions during carcinogenesis could serve as a sensitive screening tool, particularly because different methylation patterns of tumor suppressor genes have been identified in HPV-induced tumors [17]. Methylation markers are valuable in cervical cancer screening programs, with studies showing they have higher specificity compared with HPV testing and immunohistochemistry (*p16/Ki-67*) [18]. These markers can be utilized not only in tissue samples but also in any body fluid (liquid biopsy). Here are several methylation kits currently available on the market. The QIAsure Methylation Test (Qiagen, Hilden, Germany) is a multiplex quantitative methylation-specific PCR (qMSP)-based assay that amplifies the methylated promoter regions of the *FAM19A4/miR-124-2* genes, showing increased sensitivity for identifying advanced transforming CIN3+ (69.4–77.8%) and cervical cancer (100%) in hrHPV-positive samples [19]. Another MSP-based assay, GynTect^®^ (Oncgnostics, Jena, Germany), distinguishes between cervical lesion types by examining the methylation status of the promoter regions of six genes (astrotactin1 (*ASTN1*), distal-less homeobox 1 (*DLX1*), integrin subunit α 4 (*ITGA4*), relaxin family peptide receptor 3 (*RXFP3*), SRY-Box Transcription Factor 17 (*SOX17*), and zinc finger protein 671 (*ZNF671*)) and uses two quality control markers (iduronate 2-sulfatase-M (*ID2S*) and acetylcholinesterase (*AChE*)). This assay showed a sensitivity for CIN3+ ranging from 31.6% to 67.7% and a specificity for <CIN3 ranging from 82.6% to 95.9% [20,21]. Other methylation tests include Confidence Marker (Neumann Diagnostics, Budapest, Hungary), Cervi-M (Ingenuity Healthcare, Mumbai, India), Precursor-M Test^®^ (Self-screen B.V., Amsterdam, The Netherlands), PAX1 DNA Detection kit, ZNF582 DNA Detection kit (iStat Biomedical Co., Ltd., New Taipei City, Taiwan) and S5 classifier (S5^®^CareLYFE, Zhuhai, China) [21]. 

Therefore, we intended to evaluate further and validate the prospective prognostic potential of two significant genes, *EIF4G3* and *SF3B1*, that we previously identified with increased mRNA expression levels in precursor lesions through lavage sample testing these markers in a cohort study (new patients) and determine the risk of progression of low- and high-grade CIN lesions [10].

The *SF3B1* gene encodes the largest subunit of the splicing factor 3b protein complex, which is essential for spliceosome assembly and mRNA splicing. When the *SF3B1* gene is mutated, it produces a protein that alters the normal mRNA processing mechanism, leading to the abnormal splicing and potential downregulation of numerous mRNAs [22]. The involvement of this factor in cervical cancer development is unknown. However, mutations in the *SF3B1* gene are the most common and significant among spliceosome mutations in hematological diseases [23]. *SF3B1* mutations are known to contribute to tumor pathogenesis by disrupting various cellular functions and pathways, including heme biosynthesis, mitochondrial metabolism, and the NF-κB pathway [24]. *SF3B1* is altered in approximately 15–20% of all myelodysplastic syndromes (MDS) patients, and this alteration increases to over 80% in MDS, specifically with ring sideroblasts (RS) [25]. The presence of an *SF3B1* mutation appears to be an early event in MDS pathogenesis, being linked to a unique gene expression profile, and is associated with a favorable prognosis and a low risk of progression to acute myeloid leukemia (AML) [26]. In contrast to MDSs, *SF3B1* mutations in myeloproliferative neoplasms (MPNs) seem to elevate the risk of fibrotic transformation [27]. *SF3B1* mutations are relatively uncommon in chronic myelomonocytic leukemia (CMML) patients, occurring in about 5–6% of cases, and similar to MDS, these mutations are associated with the RS phenotype [25,28,29]. Simmler P et al. showed that the splicing factor *SF3B1* is also frequently mutated in pancreatic ductal adenocarcinoma (PDAC), and *SF3B1^K700E^* functions as an oncogenic driver in PDAC, promoting the advancement of early-stage tumors by hindering the cellular response to the tumor-suppressive effects of *TGF-β* [30]. Popli P et al. revealed elevated *SF3B1* protein expression in human endometrial tumors and three endometrial cancer cell lines, consistent with increased expression of other splicing factors observed in various human cancers [31]. The in vitro experiments demonstrate that *SF3B1* enhances endometrial cancer cell proliferation, cell cycle progression, migration, and invasion [32]. This is the first study that indicated the potential involvement of *SF3B1* in cervical cancer development as the expression levels were significantly elevated in cervical cancer samples compared with the control group, but also in precursor lesions. In our previous study, we showed that its expression is inhibited when E6 and E7 oncogenes are silenced because of global chromatin deposition of the MBD2/MBD3 NuRD complex [10]. Notably, we observed significant hypomethylation of CpG islands in cervical cancer, which was significantly correlated with mRNA expression levels. This indicates that the gene promoter demethylation induced by hrHPV infection could be the main cause of increased gene expression levels. Given its high mutational rate, potential fusion with another gene, or overexpression, we could hypothesize its role as a proto-oncogene. In this context, we also examined the methylation status of gene promoters and found a progressive decrease from precancerous lesions to cervical cancer. Regarding the most heterogeneous group ASCUS and ASCH, we observed that the results from the methylation analysis discriminate better than the gene expression level between the different neoplasia types. 

A similar pattern to the *SF3B1* factor was found for the eukaryotic translation initiation factor 4G (*EIF4G3*) gene. *EIF4G3* is an important scaffold protein in the translation initiation complex. It is part of the *EIF4F* complex, essential for initiating protein synthesis by binding to the mRNA cap structure and recruiting the ribosome to the mRNA. In a mice study by Hu J et al., the mutation in the *EIF4G3* gene was found to lead to male infertility due to meiotic arrest at the end of the meiotic prophase [33]. Evaluating its mRNA expression levels, we concluded that they increase with the progression of precursor lesions to cervical cancer. Moreover, the investigation of gene promoters’ methylation status revealed higher percentages in control groups versus precursor lesions or cervical cancer, and the expression levels also correlated with methylation status. Studies about the role of this factor in oncogenesis are scarce; therefore, this study is the first to report the involvement in cervical oncogenesis. However, this study has a limitation in terms of the number of patients included, but it could serve as a strong foundation for a larger investigation. 

## 5. Conclusions

Measuring methylation levels alongside gene expression levels could be a valuable tool for stratifying hr-HPV-positive patients with abnormal Pap tests, especially those with LGSIL, ASCUS, and ASCH cytology. It is well known that LGSIL may either regress or progress to more advanced stages of cervical cancer, while ASCUS and ASCH citologies are highly heterogeneous. 

This study highlights the importance of epigenetic changes, particularly aberrant methylation, in the development of cervical cancer. By identifying new factors such as *SF3B1* and *EIF4G3*, which show increased mRNA expression and altered methylation patterns in cervical cancer and precursor lesions, the research underscores the potential of these genes as biomarkers for early detection and diagnostic precision. While the role of *SF3B1* in cervical cancer development is newly reported here, its involvement in other cancers and cellular processes indicates its broader oncogenic potential. Similarly, *EIF4G3*’s role in oncogenesis is underexplored, but this study establishes its relevance in cervical cancer progression. 

## Figures and Tables

**Table 1:** The sequences of primers used in this study and their parameters.

Gene Name	Primer Sequence	Amplicon Size (bp)	Temperature (°C)	Reference
*EIF4G3 M_F*	TTTTTAGTAGTTTTCGGAAAGAGTC	163	58	This study
*EIF4G3 M_R*	GATAAATTTTCTTCACTCAACGAA			
*EIF4G3 U_F*	TTTAGTAGTTTTTGGAAAGAGTTGA	163	56	
*EIF4G3 U_R*	CCAATAAATTTTCTTCACTCAACAAA			
*SF3B1 M_F*	TAAGGATTTTACGGTTCGGTTC	182	58	
*SF3B1 M_R*	CTAAAAACTACACTCTACGCGTACG			
SF3B1 U_F	TTTAAGGATTTTATGGTTTGGTTTG	182	58	
*SF3B1 U_R*	AAAAACTACACTCTACACATACACC			
*EIF4G3_F*	ACAGAATGCAGGTCCAACCA	93	60	[10]
*EIF4G3_R*	GGCCTCTGGAAAAACGGAGA			
*SF3B1_F*	AAAAGCATAGGCGGACCATGA	70	60	
*SF3B1_R*	GGGGTTTTCCCTCCATCTGC			
*GAPDH*	CCATCTTCCAGGAGCGAGATCCCT		60	
*GAPDH*	TGAGCCCCAGCCTTCTTCATGGT			

**Table 2:** Statistical parameters of promoter methylation levels for *EIF4G3* and *SF3B1* genes in studied groups.

	EIF4G3 (Mean ± SD)	*p*-Value *	SF3B1 (Mean ± SD)	*p*-Value
NILM (−)	89.36 ± 5.771	-	87.99 ± 6.160	-
ASCUS	49.59 ± 16.160	<0.0001	55.79 ± 13.770	<0.0001
LGSIL	34.20 ± 10.170	<0.0001	28.73 ± 6.110	<0.0001
ASCH	13.08 ± 8.597	<0.0001	11.66 ± 3.432	<0.0001
HGSIL	14.21 ± 6.084	<0.0001	8.19 ± 7.523	<0.0001
SCC	2.34 ± 2.296	<0.0001	1.09 ± 1.094	<0.0001

For analysis using the *t*-test, *p*-values <0.05 were considered statistically significant (* *p*-value compared with the control group NILM).

**Table 3:** Statistical parameters of *EIF4G3* and *SF3B1* gene expression in studied groups.

	EIF4G3 (Mean ± SD)	*p*-Value *	SF3B1 (Mean ± SD)	*p*-Value
NILM (−)	−4.369 ± 0.810	−	−4.201 ± 0.791	-
ASCUS	−3.458 ± 0.919	0.0545	−3.500 ± 0.351	0.0831
LGSIL	−2.305 ± 0.415	<0.0001	−2.672 ± 1.099	0.0021
ASCH	−2.935 ± 0.597	0.0011	−2.383 ± 0.686	<0.0001
HGSIL	−2.969 ± 0.677	0.0021	−2.663 ± 0.658	0.0005 **
SCC	−2.314 ± 0.675	<0.0001 **	−1.694 ± 0.668	<0.0001 **

For analysis using the *t*-test, *p*-values < 0.05 were considered statistically significant. * *p*-value compared with the control group NILM. ** Mann–Whitney test.

## Data Availability

The original contributions presented in the study are included in the article/supplementary material, further inquiries can be directed to the corresponding author.

## Supplementary Materials

The following supporting information can be downloaded at: https://www.mdpi.com/article/10.3390/reports7030071/s1, Table S1: *p*-values from the Shapiro–Wilk test when assessing normality in the studied groups. *p*-value > 0.05.

## References

[1] Bray F., Laversanne M., Sung H., Ferlay J., Siegel R.L., Soerjomataram I., Jemal A. (2024). Global cancer statistics 2022: GLOBOCAN estimates of incidence and mortality worldwide for 36 cancers in 185 countries. CA Cancer J. Clin..

[2] Steenbergen R.D., Snijders P.J., Heideman D.A., Meijer C.J. (2014). Clinical implications of (epi)genetic changes in HPV-induced cervical precancerous lesions. Nat. Rev. Cancer.

[3] Sadia H., Shahwani I.M., Bana K.F.M. (2022). Risk factors of cervical cancer and role of primary healthcare providers regarding PAP smears counseling: Case control study. Pak. J. Med. Sci..

[4] Zhu H., Zhu H., Tian M., Wang D., He J., Xu T. (2020). DNA Methylation and Hydroxymethylation in Cervical Cancer: Diagnosis, Prognosis and Treatment. Front. Genet..

[5] Fang J., Zhang H., Jin S. (2014). Epigenetics and cervical cancer: From pathogenesis to therapy. Tumour Biol..

[6] Hesselink A.T., Heideman D.A., Steenbergen R.D., Coupé V.M., Overmeer R.M., Rijkaart D., Berkhof J., Meijer C.J., Snijders P.J. (2011). Combined promoter methylation analysis of CADM1 and MAL: An objective triage tool for high-risk human papillomavirus DNA-positive women. Clin. Cancer Res..

[7] Eijsink J.J., Lendvai Á., Deregowski V., Klip H.G., Verpooten G., Dehaspe L., de Bock G.H., Hollema H., van Criekinge W., Schuuring E. (2012). A four-gene methylation marker panel as triage test in high-risk human papillomavirus positive patients. Int. J. Cancer.

[8] Botezatu A., Goia-Rusanu C.D., Iancu I.V., Huica I., Plesa A., Socolov D., Ungureanu C., Anton G. (2011). Quantitative analysis of the relationship between microRNA-124a, -34b and -203 gene methylation and cervical oncogenesis. Mol. Med. Rep..

[9] Lorincz A.T. (2016). Virtues and weaknesses of DNA methylation as a test for cervical cancer prevention. Acta Cytol..

[10] Fudulu A., Diaconu C.C., Iancu I.V., Plesa A., Albulescu A., Bostan M., Socolov D.G., Stoian I.L., Balan R., Anton G. (2024). Exploring the Role of E6 and E7 Oncoproteins in Cervical Oncogenesis through MBD2/3-NuRD Complex Chromatin Remodeling. Genes.

[11] Li L.C., Dahiya R. (2002). MethPrimer: Designing primers for methylation PCRs. Bioinformatics.

[12] Pfaffl M.W. (2001). A new mathematical model for relative quantification in real-time RT–PCR. Nucleic Acids Res..

[13] Fackler M.J., McVeigh M., Mehrotra J., Blum M.A., Lange J., Lapides A., Garrett E., Argani P., Sukumar S. (2004). Quantitative multiplex methylation-specific PCR assay for the detection of promoter hypermethylation in multiple genes in breast cancer. Cancer Res..

[14] Cuzick J., Arbyn M., Sankaranarayanan R., Tsu V., Ronco G., Mayrand M.H., Dillner J., Meijer C.J. (2008). Overview of human papillomavirus-based and other novel options for cervical cancer screening in developed and developing countries. Vaccine.

[15] Cuzick J., Bergeron C., von Knebel Doeberitz M., Gravitt P., Jeronimo J., Lorincz A.T., J L M Meijer C., Sankaranarayanan R., J F Snijders P., Szarewski A. (2012). New technologies and procedures for cervical cancer screening. Vaccine.

[16] Lleras R.A., Smith R.V., Adrien L.R., Schlecht N.F., Burk R.D., Harris T.M., Childs G., Prystowsky M.B., Belbin T.J. (2013). Unique DNA methylation loci distinguish anatomic site and HPV status in head and neck squamous cell carcinoma. Clin. Cancer Res..

[17] Wentzensen N., Sherman M.E., Schiffman M., Wang S.S. (2009). Utility of methylation markers in cervical cancer early detection: Appraisal of the state-of-the-science. Gynecol. Oncol..

[18] Sahasrabuddhe V.V., Luhn P., Wentzensen N. (2011). Human papillomavirus and cervical cancer: Biomarkers for improved prevention efforts. Future Microbiol..

[19] Bonde J., Floore A., Ejegod D., Vink F.J., Hesselink A., van de Ven P.M., Valenčak A.O., Pedersen H., Doorn S., Quint W.G. (2021). Methylation markers FAM19A4 and miR124-2 as triage strategy for primary human papillomavirus screen positive women: A large European multicenter study. Int. J. Cancer.

[20] Dippmann C., Schmitz M., Wunsch K., Schütze S., Beer K., Greinke C., Ikenberg H., Hoyer H., Runnebaum I.B., Hansel A. (2020). Triage of hrHPV-positive women: Comparison of two commercial methylation-specific PCR assays. Clin. Epigenetics.

[21] Burdier F.R., Waheed D.E., Nedjai B., Steenbergen R.D.M., Poljak M., Baay M., Vorsters A., Van Keer S. (2024). DNA methylation as a triage tool for cervical cancer screening—A meeting report. Prev. Med. Rep..

[22] Yoshimi A., Abdel-Wahab O. (2017). Molecular Pathways: Understanding and Targeting Mutant Spliceosomal Proteins. Clin. Cancer Res..

[23] Visconte V., Makishima H., Maciejewski J.P., Tiu R.V. (2012). Emerging roles of the spliceosomal machinery in myelodysplastic syndromes and other hematological disorders. Leukemia.

[24] Zhou Z., Gong Q., Wang Y., Li M., Wang L., Ding H., Li P. (2020). The biological function and clinical significance of SF3B1 mutations in cancer. Biomark. Res..

[25] Malcovati L., Stevenson K., Papaemmanuil E., Neuberg D., Bejar R., Boultwood J., Bowen D.T., Campbell P.J., Ebert B.L., Fenaux P. (2020). SF3B1-mutant MDS as a distinct disease subtype: A proposal from the International Working Group for the Prognosis of MDS. Blood.

[26] Cazzola M., Rossi M., Malcovati L. (2013). Associazione Italiana per la Ricerca sul Cancro Gruppo Italiano Malattie Mieloproliferative Biologic and clinical significance of somatic mutations of SF3B1 in myeloid and lymphoid neoplasms. Blood.

[27] Zhao L.-P., Daltro de Oliveira R., Marcault C., Soret J., Gauthier N., Verger E., Maslah N., Roux B., Parquet N., Dosquet C. (2020). SF3B1 mutations in the Driver Clone Increase the Risk of Evolution to Myelofibrosis in Patients with Myeloproliferative Neoplasms (MPN). Blood.

[28] Yoshida K., Sanada M., Shiraishi Y., Nowak D., Nagata Y., Yamamoto R., Sato Y., Sato-Otsubo A., Kon A., Nagasaki M. (2011). Frequent pathway mutations of splicing machinery in myelodysplasia. Nature.

[29] Patnaik M.M., Lasho T.L., Finke C.M., Hanson C.A., Hodnefield J.M., Knudson R.A., Ketterling R.P., Pardanani A., Tefferi A. (2013). Spliceosome mutations involving SRSF2, SF3B1, and U2AF35 in chronic myelomonocytic leukemia: Prevalence, clinical correlates, and prognostic relevance. Am. J. Hematol..

[30] Simmler P., Ioannidi E.I., Mengis T., Marquart K.F., Asawa S., Van-Lehmann K., Kahles A., Thomas T., Schwerdel C., Aceto N. (2023). Mutant SF3B1 promotes malignancy in PDAC. eLife.

[31] Alsafadi S., Houy A., Battistella A., Popova T., Wassef M., Henry E., Tirode F., Constantinou A., Piperno-Neumann S., Roman-Roman S. (2016). Cancer-associated SF3B1 mutations affect alternative splicing by promoting alternative branchpoint usage. Nat. Commun..

[32] Popli P., Richters M.M., Chadchan S.B., Kim T.H., Tycksen E., Griffith O., Thaker P.H., Griffith M., Kommagani R. (2020). Splicing factor SF3B1 promotes endometrial cancer progression via regulating KSR2 RNA maturation. Cell Death Dis..

[33] Hu J., Sun F., Handel M.A. (2018). Nuclear localization of EIF4G3 suggests a role for the XY body in translational regulation during spermatogenesis in mice. Biol. Reprod..

