# Supplementary material for: Assessing the Methylation Status of Two Potential Key Factors Involved in Cervical Oncogenesis
Source: Reports (MDPI). 2024 Aug 18;7(3):71. doi: 10.3390/reports7030071 (PMC12225293; doi:10.3390/reports7030071)
Supplement: Supplementary file 1 [file reports-07-00071-s001.zip › reports-3105043-supplementary.pdf]

Supplementary Table S1. p-values from the Shapiro–Wilk test when assessing normality in the studied groups. *P*-value > 0.05

Studied groups→		NILM (-)	ASCH	ASCUS	LGSIL	HGSIL	SCC
p-value (>0.05) Shapiro-Wilk test	***Methylation*** EIF4G3	0.8294	0.3849	0.8281	0.2211	0.2493	0.0095
	Methylation SF3B1	0.9271	0.1181	0.3097	0.8515	0.0115	0.0294
	Expression EIF4G3	0.0615	0.6181	0.4994	0.5281	0.6940	0.1312
	Expression SF3B1	0.1214	0.0981	0.1500	0.0927	0.5092	0.2484
